# Increased cytotoxicity and bystander effect of 5-fluorouracil and 5**′**-deoxy-5-fluorouridine in human colorectal cancer cells transfected with thymidine phosphorylase

**DOI:** 10.1038/sj.bjc.6690589

**Published:** 1999-08

**Authors:** A Evrard, P Cuq, J Ciccolini, L Vian, J-P Cano

**Affiliations:** Laboratoire de Toxicologie du Médicament, Faculté de Pharmacie, 15 av. Charles Flahault, Montpellier, 34060 cedex 02, France; Laboratoire de Toxicocinétique et Pharmacocinétique, 27 bd. Jean Moulin, Marseille, 13385 cedex 5, France

**Keywords:** cancer gene therapy, thymidine phosphorylase, 5-FU, 5′-DFUR, bystander effect

## Abstract

5-Fluorouracil (5-FU) and 5′-deoxy-5-fluorouridine (5′-DFUR), a prodrug of 5-FU, are anticancer agents activated by thymidine phosphorylase (TP). Transfecting the human TP cDNA into cancer cells in order to sensitize them to these pyrimidine antimetabolites may be an important approach in human cancer gene therapy research. In this study, an expression vector containing the human TP cDNA (pcTP5) was transfected into LS174T human colon carcinoma cells. Eight stable transfectants were randomly selected and analysed. The cytotoxic effects of 5-FU and 5′-DFUR were higher in TP-transfected cells as compared to wild-type cells. The maximal decreases in the IC_50_ were 80-fold for 5-FU and 40-fold for 5′-DFUR. The increase in sensitivity to these pyrimidines of TP-transfected cells significantly correlated with the increase in both TP activity and TP expression. Transfected clone LS174T-c2 but not wild-type cells exhibited formation of [^3^H]FdUMP from [^3^H]5-FU. In addition the LS174T-c2 clone enhanced the cytotoxic effect of 5′-DFUR, but also that of 5-FU, towards co-cultured parental cells. For both anti-cancer agents, this bystander effect did not require cell–cell contact. These results show that both 5-FU or 5′-DFUR could be used together with a TP-suicide vector in cancer gene therapy. © 1999 Cancer Research Campaign


*British Journal of Cancer* (1999) **80**(11), 1726–1733
© 1999 Cancer Research Campaign
Article no. bjoc.1999.0589

### Increased cytotoxicity and bystander effect of 5-fluorouracil and 5′-deoxy-5-fluorouridine in human colorectal cancer cells transfected with thymidine phosphorylase

**A Evrard[1], P Cuq[1], J Ciccolini[2], L Vian[1] and J-P Cano[1]**

[1]Laboratoire de Toxicologie du Médicament, Faculté de Pharmacie, 15 av. Charles Flahault, 34060 Montpellier cedex 02, France; [2]Laboratoire de Toxicocinétique et Pharmacocinétique, 27 bd. Jean Moulin, 13385 Marseille cedex 5, France

**Summary** 5-Fluorouracil (5-FU) and 5′-deoxy-5-fluorouridine (5′-DFUR), a prodrug of 5-FU, are anticancer agents activated by thymidine phosphorylase (TP). Transfecting the human TP cDNA into cancer cells in order to sensitize them to these pyrimidine antimetabolites may be an important approach in human cancer gene therapy research. In this study, an expression vector containing the human TP cDNA (pcTP5) was transfected into LS174T human colon carcinoma cells. Eight stable transfectants were randomly selected and analysed. The cytotoxic effects of 5-FU and 5′-DFUR were higher in TP-transfected cells as compared to wild-type cells. The maximal decreases in the $IC_{50}$ were 80-fold for 5-FU and 40-fold for 5′-DFUR. The increase in sensitivity to these pyrimidines of TP-transfected cells significantly correlated with the increase in both TP activity and TP expression. Transfected clone LS174T-c2 but not wild-type cells exhibited formation of [$^3$H]FdUMP from [$^3$H]5-FU. In addition the LS174T-c2 clone enhanced the cytotoxic effect of 5′-DFUR, but also that of 5-FU, towards co-cultured parental cells. For both anti-cancer agents, this bystander effect did not require cell–cell contact. These results show that both 5-FU or 5′-DFUR could be used together with a TP-suicide vector in cancer gene therapy.

**Keywords:** cancer gene therapy; thymidine phosphorylase; 5-FU; 5′-DFUR; bystander effect

A major drawback of chemotherapy is the lack of specific toxicity towards cancer cells which leads to important side-effects that prevent the treatment from achieving tumour reduction. One strategy that could overcome this problem is to introduce into malignant cells a gene encoding an enzyme that sensitizes them to chemotherapeutic agents. Since the first experiments using the herpes simplex virus thymidine kinase (HSV-TK) (Moolten, 1986; Moolten and Wells, 1990) which activates the acyclic nucleoside analogue ganciclovir (GCV) intratumourally, various drug sensitivity genes ('suicide genes') have been reported (Mullen et al, 1992; Huber et al, 1993; Wei et al, 1994; Chen et al, 1995; Parker et al, 1997).

A critical point of this strategy is the limiting gene transfer efficiency to the tumour mass. However, the ability of a toxic anabolite, metabolically converted in the transfected cells, to diffuse into neighbouring untransfected cells could alleviate the problem. This 'bystander effect' has been observed in many cancer gene therapy experiments, and reduction of tumour mass was observed even when a small percentage of cancer cells were genetically modified (Freeman et al, 1993; Chen et al, 1995). The bystander effect is achieved by diffusion of phosphorylated nucleosides through gap junctions in the HSV-TK/ganciclovir system (Fick et al, 1995). However, experiments with other suicide genes, such as cytosine deaminase which cleaves 5-fluorocytosine to 5-fluorouracil (5-FU), have shown the advantages of a diffusible, gap junction-independent bystander effect (Huber et al, 1994; Trinh et al, 1995; Denning and Pitts, 1997).

*Received 19 October 1998*
*Revised 21 January 1999*
*Accepted 28 January 1999*

*Correspondence to:* P Cuq

Thymidine phosphorylase (EC 2.4.2.4) (TP), also described as the angiogenic platelet-derived endothelial cell growth factor (PD-ECGF) (Ishikawa et al, 1989; Moghaddam and Bicknell, 1992; Sumizawa et al, 1993; Miyadera et al, 1995), is a homodimeric enzyme with a monomeric molecular mass of about 55 kDa (Desgranges et al, 1981; Miyazono et al, 1987) and which phosphorolytically cleaves thymidine to yield thymine and deoxyribose 1-phosphate (Friedkin and Roberts, 1953; Krenitsky, 1968). TP is expressed in various human cells and tissues and plays a role in plasma thymidine homeostasis (Zimmerman and Seidenberg, 1964; Shaw et al, 1988; Fox et al, 1995). The levels of expression in different human tissues can vary up to 15-fold (Yoshimura et al, 1990). Moreover, TP levels are increased in several types of malignant tumours when compared to the non-neoplastic regions of these tissues (Obrien et al, 1996), and also in the plasma from tumour-bearing animals and cancer patients (Luccioni et al, 1994).

TP is also a key enzyme in the metabolic activation of fluoropyrimidines that share the physiological pathway of pyrimidines. TP is responsible for the conversion of 5′-deoxy-5-fluorouridine (5′-DFUR, doxifluridine) to 5-FU and, because of its reversible phosphorolytic activity, assumes also the conversion of 5-FU to its anabolite 5-fluoro-2′-deoxyuridine (5-FdUrd). Transfection experiments have provided evidence that TP mediates the sensitivity of HT-29 human colon carcinoma cells to 5-FU (Schwartz et al, 1995), and of MCF-7 human breast cancer cells to 5′-DFUR (Patterson et al, 1995). Furthermore, the induction of TP expression by interferon increases 5-FU cytotoxicity (Schwartz et al, 1994, 1998) and recent studies suggest that the level of TP activity could be a prognostic marker for the 5-FU cytotoxicity in vitro and in vivo (Fox et al, 1997; Griffiths and Stratford, 1997; Mader et al, 1997). In a recent paper, we have demonstrated an increased 5-FU sensitivity of a murine adenocarcinoma cell line in vitro and in vivo after transfection of TP cDNA (Evrard et al, 1999). Therefore, TP gene transfer in cancer cells in order to enhance their sensitivity to fluoropyrimidines could be a promising strategy in cancer gene therapy.

In this report, we investigated the effect of TP overexpression in human LS174T colorectal cancer cells on [$^3$H]5-FU metabolism and on 5-FU and 5′-DFUR sensitivity. We next examined the bystander effect on neighbouring untransfected cells in contact and non-contact conditions.

### MATERIALS AND METHODS

### Chemicals

5-FU, 5′-DFUR, enhanced chemiluminescence (ECL) reagents (luminol, hydrogen peroxide and coumaric acid) and high-performance liquid chromatography (HPLC) reagents were from Sigma (St Quentin Fallavier, France). [H$^3$]5-FU (12.5 Ci mmol$^{-1}$) was from Du Pont de Nemours (Les Ulis, France).

### Cell lines

The human colorectal adenocarcinoma LS174T cells (ATCC number, CL-188) were cultured at 37°C in a fully humidified 5% carbon dioxide atmosphere in RPMI-1640 medium supplemented with 10% fetal calf serum (FCS) and 2 mM glutamine. COS-7 cells were cultured in Dulbecco’s modified Eagle’s medium (DMEM) containing 5% FCS and 2 mM glutamine.

### Human TP cDNA subcloning

The TP cDNA was kindly provided by Dr Rangana Choudhuri (Imperial Cancer Research Fund, University of Oxford, UK). The full-length human TP cDNA was obtained by digesting the parental pPL5 vector with *Xba*I and *Hin*dIII and the insert was ligated into the same sites of the mammalian expression vector pcDNA3 (Invitrogen, NV Leek, The Netherlands) to produce pcTP5. This vector was then transformed into cells (*Escherichia coli*, DH5α) and the plasmid DNA purified using a Qiagen Plasmid Kit (Qiagen, Courtaboeuf, France).

### Transfection of human TP cDNA in COS-7 cells and LS174T cells

COS-7 and LS174T cells (1 × 10$^6$ per 20 cm$^2$ Petri dish) were cultured for 24 h until cells were approximately 80% confluent. They were then transfected with pcTP5 or pcDNA3 (control) using the Lipofectamine™ reagent (Life Technologies, Cergy-Pontoise, France) using conditions as recommended by the manufacturer. After 48 h incubation, COS-7 cells were lysed and assayed for TP activity and expression. Stable LS174T/pcTP5 transfectants were selected for geneticin (G418 sulphate, Life Technologies, Cergy-Pontoise, France) resistance (250 μg ml$^{-1}$ culture medium), and eight clones were randomly selected and analysed for 5-FU and 5′-DFUR sensitivity, TP activity and protein expression.

### Cell lysis

Cells (COS-7 or LS174T) were homogeneized on ice in 100 μl of a lysis buffer containing 50 mM Tris–HCl (pH 8.0), 150 mM sodium chloride, 100 μg ml$^{-1}$ phenylmethylsulphonyl fluoride (PMSF), 1 μg ml$^{-1}$ aprotinin and 1% Triton X-100. The lysates were centrifuged at 20 000 ***g*** for 30 min at 4°C and protein level of the supernatants was determined according to Bradford (1976).

### Immunoblotting and immunocytostaining

The mouse monoclonal IgG1 654-1 raised against human TP was kindly provided by Nippon Roche Research Center (Nishida et al, 1996) and was used as primary antibody for either immunoblotting or immunocytostaining experiments. For immunoblotting, supernatant proteins (20 μg) of cell lysates, obtained as described above, were separated on sodium dodecyl sulfate polyacrylamide gel electrophoresis (SDS-PAGE). The proteins were electroblotted onto nitrocellulose membrane, and incubated with 0.2 μg ml$^{-1}$ of primary antibody followed by horseradish peroxidase-conjugated goat anti-mouse IgG (Sigma, St Quentin Fallavier, France). Immunolabelled proteins were vizualized using X-ray film after incubation with ECL reagents. For immunocytostaining, cells were seeded on cover-slips in 12-well plates. After fixing with methanol and permeabilization with phosphate-buffered saline (PBS)–0.1% Triton, cells were incubated with 1 μg ml$^{-1}$ of primary antibody followed by fluorescein isothiocyanate (FITC)-conjugated goat anti-mouse IgG (Sigma, St Quentin Fallavier, France).

### TP activity assay

The enzyme activity in cell lysates was assayed as previously described (Yoshimura et al, 1990), with minor modifications. Briefly, 50 μl of supernatants were added to 150 μl of a reaction mixture consisting of 10 mM thymidine, 10 mM $KH_2PO_4$ (pH 8.4) and then incubated at 37°C for 4 h. The reaction was stopped by the addition of 800 μl of 0.2 N sodium hydroxide. The absorbance at 300 nm was determined and the amount of thymine in the reaction mixture was calculated using a calibration curve. The TP activity was expressed as pmol thymine$^{-1}$ μg protein$^{-1}$ h$^{-1}$.

### [$^3$H]5-FU metabolism analysis

Cells (2 × 10$^6$) were seeded in a 25 cm$^2$ flask and were treated for 4 h with 100 μCi of [$^3$H]5-FU (corresponding to a final concentration of 1.6 μM). Cells were then harvested and vortexed in 60% methanol. After methanol evaporation, the mobile phase (100 μl) was added to the residue for HPLC injection. Chromatographic conditions were as follows: reverse phase column, Licrosphere C8, 5 μm; mobile phase, buffer pH 7 10% (1.7 mM $K_2HPO_4$, 5 mM tetrabutylammonium nitrate): methanol 90%; flow rate 1 ml min$^{-1}$; isocratic conditions. Detection was performed using a radiometric detector (Flo-One Beta Radiomatic, Packard Instrument SA) and peaks identified by comparing retention times with standards.

### In vitro cytotoxicity assay

The effect of anticancer agents (5-FU, 5′-DFUR) on cell viability was assessed using the neutral red assay as previously described (Evrard et al, 1996). Briefly, aliquots of cell suspension (5 × 10$^3$ cells per well) were seeded in 96-well microtitre plates which were incu-

*© 1999 Cancer Research Campaign* 

bated for 24 h at 37°C in a fully humidified atmosphere of 5% carbon dioxide in air. The cells were then incubated for 72 h in the absence (control) or the presence of different drug concentrations (150 μl in fresh medium per well, eight wells per agent concentration). Thereafter, cells were washed with PBS and 150 μl of a neutral red solution (40 μg $ml^{-1}$) was added. After 3 h at 37°C, 5% carbon dioxide, the cells were washed with PBS and destained with 150 μl of glacial acetic acid (1%)–ethanol (50%) (v/v). Absorbances at 540 nm ($A_{540}$) were measured using a microplate reader (Labsystems Multiscan MS). The effect of the drugs on cell survival was expressed as a growth ratio calculated using the equation $A_{540}$ drug-treated/$A_{540}$ control.

### Assessment of in vitro bystander effect

TP-transfected LS174T-c2 cells and parental cells were mixed in various ratios [c2 cells/(c2 cells + parental cells)] and seeded $5 \times 10^3$ cells $ml^{-1}$ in 96-well plates. After 24 h at 37°C, the cells were incubated for 3 days in the absence (control) or the presence of different drug concentrations (0.01–100 μM) and the neutral red uptake assay was performed as described above. The results are expressed as a growth ratio relative to drug-free controls. To study the requirement of direct cell–cell contact for the bystander effect, parental LS174T cells were seeded in the bottom chamber of 96-well microtitre plates ($5 \times 10^3$ cells per well) and parental or LS174T-c2 cells ($5 \times 10^3$ cells per insert) were placed in the top chamber of membrane culture inserts (Anopore membrane, pore size 0.02 μM, Nunc, France). After a 24 h incubation at 37°C, cells

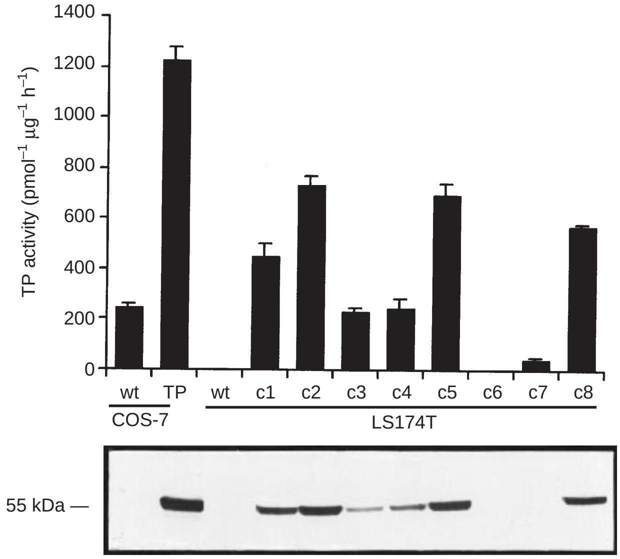

**Figure 1** Human thymidine phosphorylase expression and activity in COS-7 and LS174T cells. Upper panel: lysates from parental (wt) or pcTP5-transfected (TP) COS-7 cells, and from parental (wt) or pcTP5-transfected LS174T cell clones (c1 to c8) were incubated at 37°C for 4 h in the presence of 10 mM thymidine. The 300 nm absorbance and the amount of thymine were calculated as described above; the TP activity was expressed as pmol thymine μg $protein^{-1}$ $h^{-1}$. Data are means ± s.e.m. of three separate experiments. Lower panel: proteins from COS-7 and LS174T cell lysates were separated by SDS-PAGE and electroblotted onto nitrocellulose sheets. The human TP immunoreactivity was assessed using anti-human TP IgG

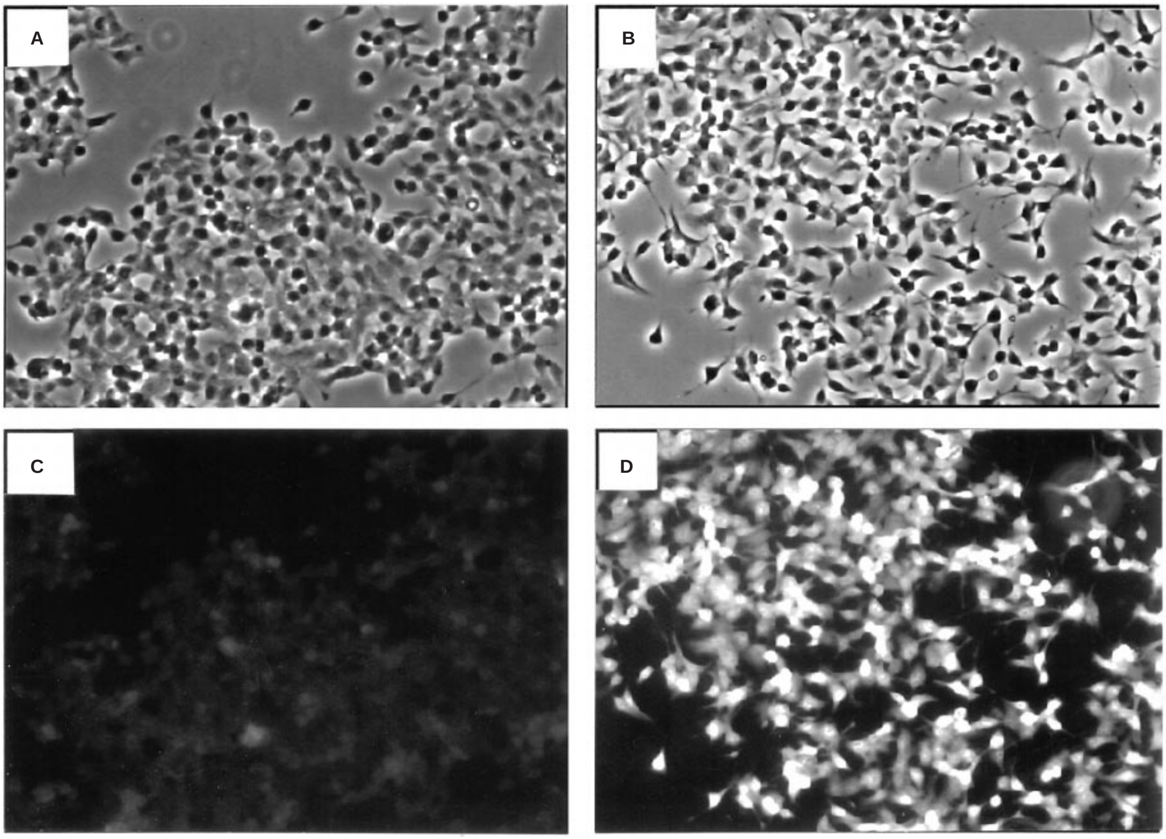

**Figure 2** Immunocytostaining of human TP in LS174T cells. Immunocytostaining was performed using mouse monoclonal IgG raised against human TP as described in Materials and Methods. Phase contrast microscopy: (**A**) parental LS174T cells, (**B**) LS174T-c2 cells; fluorescence microscopy: (**C**) parental LS174T cells, (**D**) LS174T-c2 cells. Magnification × 200

 *© 1999 Cancer Research Campaign*

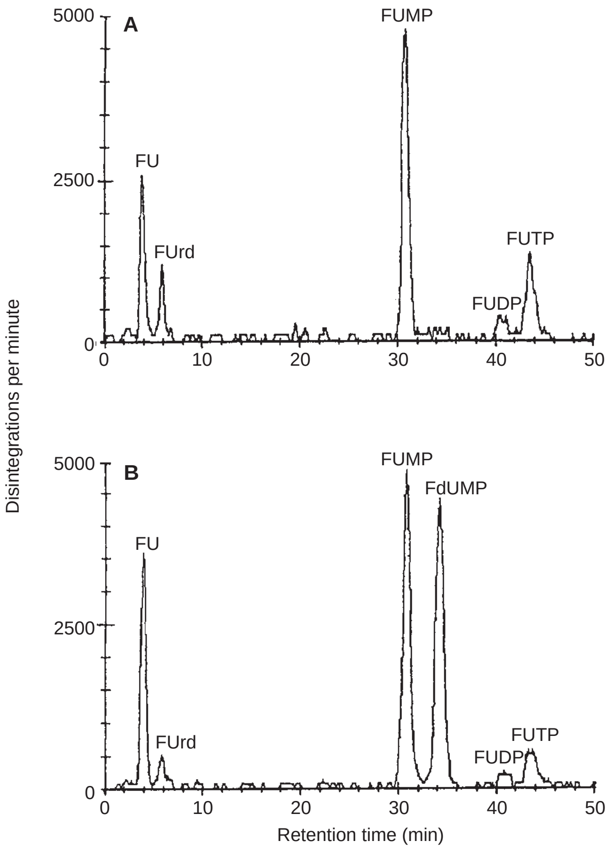

**Figure 3** Metabolism of [$^3$H]5-FU in LS174T cells. Parental (**A**) or clone 2 (**B**) LS174T cells were incubated for 4 h in the presence of 100 µCi [$^3$H]5-FU (final concentration: 1.6 µM) and the resulting metabolites were identified by HPLC coupled to a radiometric detection. Data are from one representative experiment were treated for 72 h with different concentrations of 5-FU or 5′-DFUR (0.1–100 µM) which were added into the membrane culture inserts. Thereafter, the membrane culture inserts were removed and the survival of parental cells seeded in the microtitre plates was measured as described above.

### RESULTS

### Subcloning of the human TP cDNA

In order to transfect the human TP cDNA in mammalian tumour cells, we subcloned the full-length cDNA in the mammalian expression vector pcDNA3 in a sense orientation with respect to the CMV promoter. The functionality of pcTP5 was assessed by studying the protein expression and the enzyme activity in lysates from COS-7 cells transfected with the plasmid. As shown in Figure 1, a strong and specific production of immunodetectable human TP and a high TP activity were evident in pcTP5-transfected cell lysates. An endogeneous TP activity was found in wild-type COS-7 cells but the protein was not detected because the anti-TP antibody is specific for human and not simian TP.

### Expression of human TP in LS174T cells

Human adenocarcinoma LS174T cells were transfected with the pcTP5 and stable transfectants were selected for geneticin resistance.

**Table 1** Sensitivity to 5-FU and 5′-DFUR of LS174T cells wild-type and TP-transfected clones

| | 5-FU | | 5′-DFUR | |
|---|---|---|---|---|
| **LS174T** | **$IC_{50}$ (µM)** | **-Fold** | **$IC_{50}$(µM)** | **-Fold** |
| Wild-type | 25.33 ± 0.87 | 1.0 | 10.92 ± 0.84 | 1.0 |
| pcDNA3 | 25.67 ± 0.23[a] | 1.0 | 10.52 ± 0.76[a] | 1.0 |
| Clone 1 | 0.68 ± 0.11 | 37.2 | 0.84 ± 0.08 | 13.0 |
| Clone 2 | 0.32 ± 0.01 | 79.2 | 0.27 ± 0.03 | 40.4 |
| Clone 3 | 4.69 ± 0.87 | 5.4 | 3.54 ± 0.71 | 3.1 |
| Clone 4 | 2.12 ± 0.19 | 11.9 | 2.36 ± 0.23 | 4.6 |
| Clone 5 | 0.29 ± 0.03 | 87.3 | 0.30 ± 0.03 | 36.4 |
| Clone 6 | 25.94 ± 4.43[a] | 1.0 | 9.84 ± 0.73[a] | 1.1 |
| Clone 7 | 9.36 ± 1.26 | 2.7 | 8.45 ± 1.18[a] | 1.3 |
| Clone 8 | 0.59 ± 0.07 | 42.9 | 0.65 ± 0.07 | 16.8 |

[a]Not significantly different when compared to wild-type cells, other values are significant with $P < 0.01$ (Student's *t*-test). $IC_{50}$s were determined using the neutral red assay. Each value represents the mean ± s.e.m. of three separate experiments.

The growth rate of the transfected LS174T cells did not significantly vary from that of the wild-type cells or that of pcDNA3-transfected cells (data not shown). Eight clones of pcTP5-transfected cells (c1 to c8) were randomly selected and analysed for TP activity and expression. The levels of enzyme activity, ranging from 0 to 730 $pmol^{-1}$ $\mu g^{-1}$ $h^{-1}$, were closely related to protein expression as shown by Western blot experiments (Figure 1). The parental cells expressed neither immunodetectable TP nor significant TP activity. Clone 2 (LS174T-c2), which exhibited the highest TP protein expression and activity (Figures 1 and 2), was used for analysis of the bystander effect and [$^3$H]5-FU metabolism.

### [$^3$H]5-FU metabolism

The increase expression of functional TP in LS174T-c2 cells would lead to a greater metabolic activation of 5-FU to deoxyribonucleotides. To address this, parental and LS174T-c2 cells were incubated in the presence of [$^3$H]5-FU and the resulting metabolites were identified by HPLC coupled to a radiometric detection. As shown in Figure 3, a significant amount of [$^3$H]5-fluorodeoxyuridine-monophosphate (5-dFUMP) accumulated in LS174T-c2 cells but not in parental cells, whereas similar levels of [$^3$H]ribonucleotides (FUrd, FUMP, FUDP, FUTP) were detected in both cell lines. Thus, TP-transfected cells displayed [$^3$H]5-FU activation through both desoxyribonucleotides and ribonucleotides pathways, whereas [$^3$H]5-FU was exclusively activated through the ribonucleotides pathway in parental cells.

### In vitro cytotoxicity

The effect of human TP expression on the sensitivity of LS174T cells to 5-FU and 5′-DFUR was studied by using the neutral red uptake assay. 5-FU and 5′-DFUR decreased the cell viability in a concentration-dependent fashion. The results expressed as $IC_{50}$ are shown in Table 1. The sensitivity to the fluoropyrimidines of LS174T cells transfected with pcDNA3 was not significantly different from that of parental cells. On the other hand, TP-expressing LS174T cells were more sensitive to the cytotoxic actions of 5-FU and 5′-DFUR than the parental cells. Clones 2 and

© 1999 Cancer Research Campaign

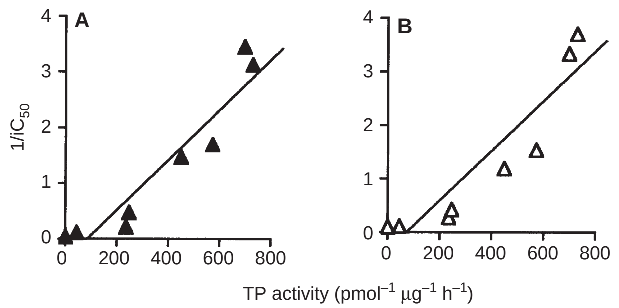

**Figure 4** Correlation between TP activity and 5-FU (**A**) or 5′-DFUR (**B**) cytotoxicity in TP-transfected LS174T cell clones. TP activity and 1/$IC_{50}$ were significantly correlated for both 5-FU ($r^2$ = 0.9) and 5′-DFUR ($r^2$ = 0.85). Data are means of three separate experiments

5 were found to be approximately 80-fold and 40-fold more sensitive to 5-FU and 5′-DFUR respectively. Moreover, there was a significant correlation between the TP activity of the cell clones and their in vitro sensitivity to 5-FU and 5′-DFUR ($r^2$ = 0.9 and 0.85 respectively) (Figure 4A,B).

### Bystander effect

Mixed cultures of parental and TP-transfected cells were generated to study the involvement of a bystander effect in the in vitro cytotoxicity of 5-FU and 5′-DFUR. When parental LS174T cells were co-cultured with LS174T-c2 cells at a ratio of 0.1, the $IC_{50}$ values of 5-FU and 5′-DFUR were lowered approximately tenfold, indicating a clear bystander effect (Figure 5 A, B). There were significant bystander effects (50% cell death) for both anticancer agents (5 µM final concentration) even when only 5% of cells were transfected (Figure 5 C, D).

We next investigated whether this bystander effect would require direct cell–cell contact. The sensitivity to 5-FU of parental cells was significantly higher when they were co-cultured, as described above, with LS174T-c2 cells rather than with parental cells in the culture inserts (Figure 6A). Similar results were obtained with 5′-DFUR (Figure 6B).

### DISCUSSION

Despite the extended use of fluoropyrimidines, in particular 5-FU, in advanced colorectal cancer, response rates are only 10–20%

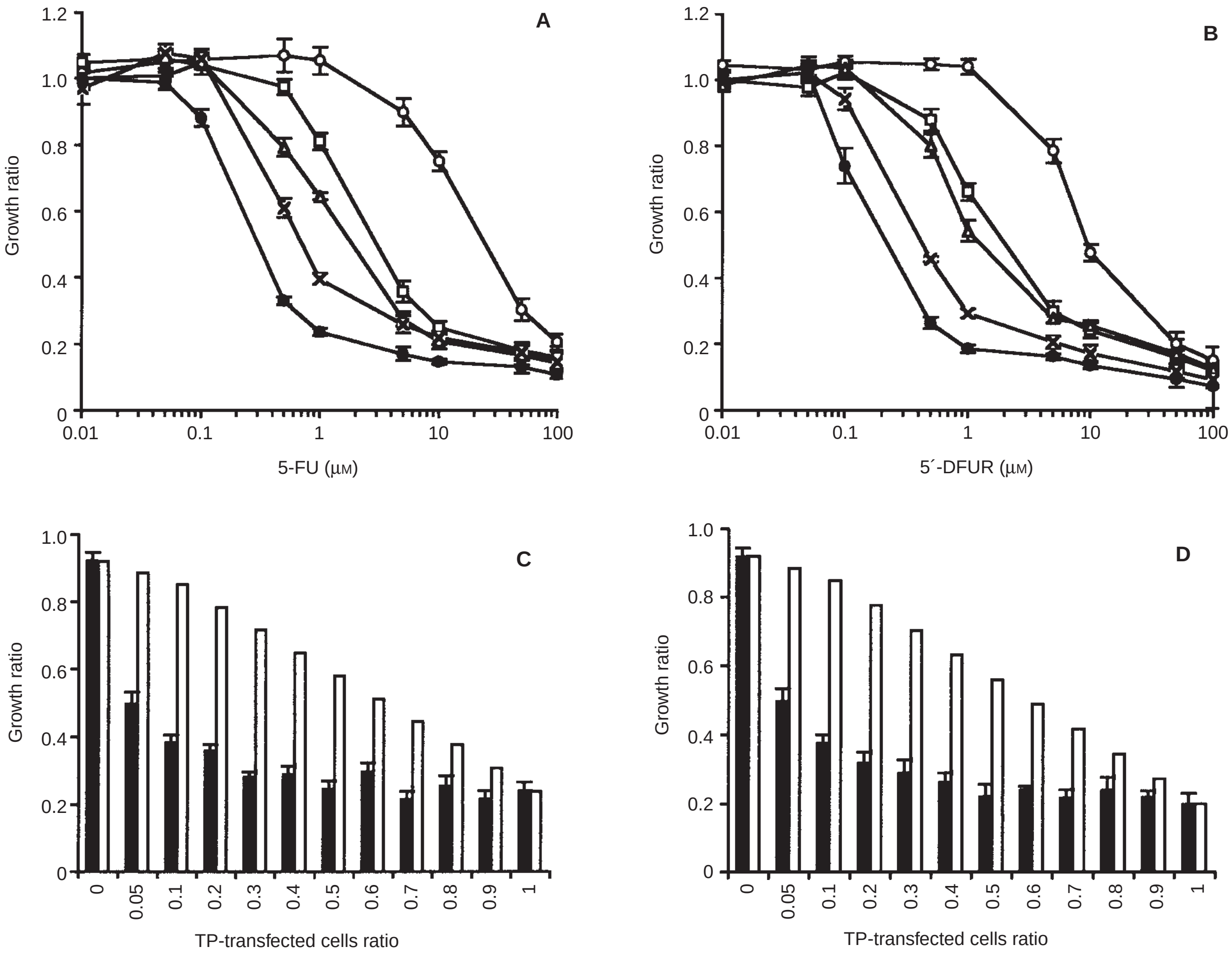

**Figure 5** Bystander effect of 5-FU and 5′-DFUR. The cell viability was studied by using the neutral red assay. Upper panel: TP-transfected LS174T cells (clone 2) were mixed and co-cultured with parental cells at various ratio (○ = 0, □ = 0.1, △ = 0.2, × = 0.5, ● = 1) and then incubated in the presence of 5-FU (**A**) or 5′-DFUR (**B**). Lower panels: Effect of 5 µM 5-FU (**C**) or 5′-DFUR (**D**) on co-cultured parental and LS174T-c2 cells at various ratios. Open bars, theoretical growth ratio; solid bars, observed growth ratio. Data are means ± s.e.m. of eight wells from a microtiter plate column

© 1999 Cancer Research Campaign

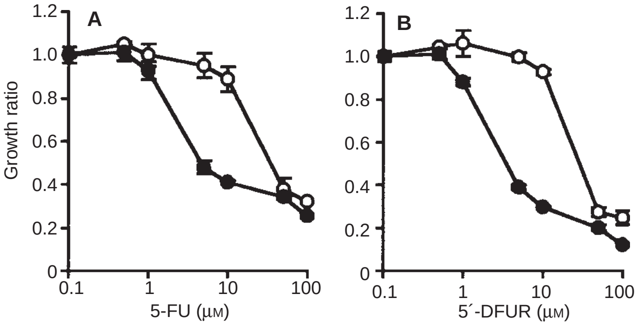

**Figure 6** Diffusibility of the bystander effect. Parental and TP-transfected (clone 2) LS174T cells were co-cultured in non-contact conditions as described above. The viability of parental cells, seeded on the lower chamber and co-cultured with parental cells (○) or TP-transfected cells (●) in the culture inserts, was assessed using the neutral red assay after exposure to 5-FU (**A**) or 5′-DFUR (**B**). Data are means ± s.e.m. of eight wells from a microtiter plate column

(Cohen et al, 1993). Different strategies, including combination of 5-FU with other cytotoxic agents or with cytokines, are invoked to increase the sensitivity of cancer cells to fluoropyrimidines (Elias and Crismann, 1988; Schwartz et al, 1994). A new gene transfer approach is based on the fact that 5-FU and 5′-DFUR, a prodrug of 5-FU, are activated by TP. Thus, transferring the gene encoding human TP into cancer cells could render them more susceptible to 5-FU and/or 5′-DFUR. In this report, we examined the effects of TP gene transfer in human colon cancer cells LS174T and their subsequent sensitivity to fluoropyrimidines.

The human colon adenocarcinoma cell line LS174T was chosen for establishing stable transfectants. Neither TP activity nor TP expression were detectable in parental LS174T cells. Therefore, LS174T cell line was an ideal model for studying the effects of TP overexpression on colonic cancer cells sensitivity to fluoropyrimidines. From the selected TP-transfected cell clones, we observed a good correlation between protein expression and TP activity levels. Clone LS174T-c2, which exhibited the highest TP activity, was chosen for [$^3$H]5-FU metabolic profile analysis and bystander effect experiments.

5-FU has been reported to be activated through two distinct pathways (Rustum et al, 1997; Sobrero et al, 1997). The first, initiated by 5-FU conversion to 5-fluorouridine (FUrd) by uridine phosphorylase (UP), leads to fluororibonucleotides incorporation into RNA. The second, initiated by 5-FU conversion to 5-fluoro-2′-deoxyuridine (FdUrd) by thymidine phosphorylase, leads to thymidylate synthase inhibition by 5-fluoro-2′-deoxyuridine monophosphate (FdUMP) and to fluorodeoxyribonucleotides incorporation into DNA. HPLC analysis of [$^3$H]5-FU metabolism was carried out to evaluate the relative importance of the two pathways in parental LS174T and LS174T-c2 cells. Parental LS174T cells displayed an accumulation of [$^3$H]fluororibonucleotides (FUrd, FUMP, FUDP, FUTP) reflecting the exclusive activation of [$^3$H]5-FU by the ribonucleotides pathway. In agreement with the metabolic profile, parental LS174T cells did not express thymidine phosphorylase activity whereas a significant endogeneous uridine phosphorylase activity was detected (data not shown). In contrast, beside the synthesis of [$^3$H]fluororibonucleotides, a strong formation of [$^3$H]FdUMP was detected in TP-transfected LS174T-c2 cells. Similarly, Shwartz et al (1995) reported a correlation of TP activity with FdUMP levels in human colon carcinoma HT-29 cells. The first anabolite after 5-FU activation by TP, i.e. [$^3$H]FdUrd, was not detected in transfected cells, suggesting that the conversion of FdUrd to FdUMP by thymidine kinase is not a limiting step and occurs rapidly. This result, as previously suggested (Mader et al, 1997), argues against a decisive role of thymidine kinase in the activation of 5-FU. In conclusion, these data clearly indicate that TP is able to activate 5-FU when overexpressed in LS174T cancer cells.

We next assessed the in vitro sensitivity to 5-FU and 5′-DFUR of parental and TP-transfected LS174T cells. The cytotoxicity of both 5-FU and 5′-DFUR was enhanced by TP expression; cell sensitivity and TP activity were correlated highly. The maximal decreases in the $IC_{50}$ values observed with LS174T-c2 cells were 80-fold for 5-FU and 40-fold for 5′-DFUR. Such a significant increase of both fluoropyrimidines cytotoxic effects by TP gene transfer into human cancer cells has never been reported so far. Schwartz et al (1995) reported a lower enhancement (19-fold) of human colon cancer cells (HT-29) sensitivity to 5-FU by TP transfection. Other works have shown that TP transfection in MCF-7 (Patterson et al, 1995) or PC-9 (Kato et al, 1997) cells increased the sensitivity to 5′-DFUR but not to 5-FU. This discrepancy could be explained by a higher sensitivity of the parental cells to 5-FU correlated to an endogenous TP activity. Therefore, parental LS174T cells which exhibit no TP activity were relatively resistant to 5-FU despite activation of 5-FU by UP. Activation of the desoxyribonucleotides pathway by TP overexpression in LS174T cells greatly enhanced 5-FU cytotoxicity. Our data are consistent with previous reports proposing TP as a biochemical determinant of response to 5-FU and suggest that the loss of TP activity could be responsible for 5-FU resistance (Chu et al, 1990; Schwartz et al, 1995; Fox et al, 1997; Mader et al, 1997).

By contrast, parental LS174T displayed high sensitivity to 5′-DFUR when compared to PC-9 (Kato et al, 1997) or KB (Haraguchi et al, 1993) cancer cell lines. Since LS174T cells express significant UP activity but no TP activity, 5′-DFUR may be efficiently activated by the endogeneous UP. After TP transfection, LS174T-c2 clones were found to be 40-fold more sensitive to 5′-DFUR than parental cells. This result strongly suggests that metabolic activation of 5′-DFUR is achieved by both TP and UP in cancer cells. Therefore, a cancer gene therapy approach using 5′-DFUR as prodrug and TP as a suicide gene should account for basal TP activity and also basal UP activity of tumour cells.

The increased sensitivity to 5-FU and 5′-DFUR of TP transfected LS174T cells has led us to examine the neighbouring cytotoxicity on adjacent, untransfected cells. In agreement with previous reports (Patterson et al, 1995; Kato et al, 1997), we describe here a bystander effect with 5′-DFUR and, moreover, we demonstrate for the first time a bystander effect with 5-FU. In our experiments, a TP-transfected cell ratio of 0.05 is sufficient to obtain 50% cell death and a ratio of 0.2–0.3 is sufficent for a maximal effect on the whole cell population in the presence of 5 μM 5-FU or 5′-DFUR.

Depending on the suicide gene, the bystander effect can be achieved by diffusion of activated anabolites from transfected to untransfected cells in a cell–cell contact-dependent (Fick et al, 1995) or -independent (Huber et al, 1994; Chen and Waxman, 1995; Kato et al, 1997) fashion. [$^3$H]5-FU metabolic profile analysis demonstrated high level of FdUMP in TP-transfected but not in parental LS174T cells. However, this phosphorylated compound is not diffusible and a bystander effect could occur through gap junctions as is the case for phosphorylated derivatives of ganciclovir. As 5-FdUrd

*© 1999 Cancer Research Campaign* 

is able to cross the cell membrane through an in-and-out nucleoside transport (Grem and Fisher, 1986; Lonn et al, 1989) we suggest that 5-FdUrd, although not detected in HPLC experiments, could be responsible for the diffusible bystander effect observed in cytotoxicity assays in non-contact conditions.

We are aware of the angiogenic effect of TP and of the potential resulting tumour growth enhancement. However, we think that it does not compromise its use as a suicide gene because TP-transfected cells would not be able to promote angiogenesis in so far as they would be selectively killed by the fluoropyrimidine chemotherapy. On the other hand, a transient expression of TP in target cells could overcome this problem.

In conclusion, our data suggest that level of TP activity is strongly implicated in the 5′-DFUR but also 5-FU sensitivity of human colon carcinoma cells. Therefore TP should be considered as a potential suicide gene for cancer gene therapy with 5-FU or 5′-DFUR. High gene transfer efficiency and specific targeting of TP gene in tumour cells could be achieved by using recombinant viral vectors and promoter elements of genes usually transcribed by tumours such as carcinoembryonic antigen in colon carcinoma. Furthermore, our results have a direct clinical relevance, especially since a number of 5-FU prodrugs such as capecitabine, activated by TP, are in clinical trials. Thus, these data should provide a basis for measuring TP in clinical material and correlate with outcome of fluoropyrimidine-based therapy.

### AKNOWLEDGEMENTS

The authors are grateful to Paul Bello for reading the manuscript. This work was supported by grants from Association pour la Recherche sur le Cancer, Ligue Nationale contre le Cancer and Groupement des Entreprises Françaises dans la Lutte contre le Cancer.

### REFERENCES

Bradford MM (1976) A rapid and sensitive method for the quantitation of microgram quantities of protein utilizing the principle of protein-dye binding. *Anal Biochem* **72**: 248–254

Chen CY, Chang YN, Ryan P, Linscott M, McGarrity GJ and Chiang YL (1995) Effect of herpes simplex virus thymidine kinase expression levels on ganciclovir-mediated cytotoxicity and the ‘bystander effect’. *Hum Gene Ther* **6**: 1467–1476

Chen L and Waxman DJ (1995) Intratumoral activation and enhanced chemotherapeutic effect of oxazophosphorines following cytochrome P-450 gene transfer: development of a combined chemotherapy/cancer gene therapy strategy. *Cancer Res* **55**: 581–589

Chu E, Lai GM, Zinn S and Allegra CJ (1990) Resistance of a human ovarian cancer line to 5-fluorouracil associated with decreased levels of 5-fluorouracil in DNA. *Mol Pharmacol* **38**: 410–417

Cohen AM, Minsky BD and Schilsky RL (1993) Colon cancer. In: *Cancer, Principles and Practices of Oncology*, 4th edn, Devita VT, Hellman S and Rosenberg SA (eds), pp. 929–977. JB Lippincott: Philadelphia

Denning C and Pitts JD (1997) Bystander effect of different enzyme-prodrug systems for cancer gene therapy depend on different pathways for intercellular transfer of toxic metabolites, a factor that will govern clinical choice of appropriate regimes. *Hum Gene Ther* **8**: 1825–1835

Desgranges C, Razaka G and Rabaud H (1981) Catabolism of thymidine in human blood platelets – purification and properties of thymidine phosphorylase. *Biochim Biophys Acta* **654**: 211–218

Elias L and Crismann HA (1988) Interferon effects upon the adenocarcinoma 38 and HL-60 cell lines: antiproliferative responses and synergistic interactions with halogenated pyrimidine antimetabolites *Cancer Res* **48**: 4868–4873

Evrard A, Vian L, Aubert C and Cano JP (1996) An in vitro nucleoside analog screening method for cancer gene therapy. *Cell Biol Toxicol* **12**: 345–350

Evrard A, Cuq P, Robert B, Vian L, Pèlegrin A and Cano JP (1999) Enhancement of 5-fluorouracil cytotoxicity by human thymidine phosphorylase expression in cancer cells: in vitro and in vivo study. *Int J Cancer* **80**: 465–470

Fick J, Barker FG II, Dazin P, Westphale EM, Beyer EC and Israel MA (1995) The extent of heterocellular communication mediated by gap junctions is predictive of bystander tumor cytotoxicity in vitro. *Proc Natl Acad Sci USA* **92**: 11071–11075

Fox SB, Moghaddam A, Westwood M, Turley H, Bicknell R, Gatter KC and Harris AL (1995) Platelet-derived endothelial cell growth factor thymidine phosphorylase expression in normal tissues – an immunohistochemical study. *J Pathol* **176**: 183–190

Fox SB, Engels K, Comley M, Whitehouse RM, Turley H, Gatter KC and Harris AL (1997) Relationship of elevated tumour thymidine phosphorylase in node-positive breast carcinomas to the effects of adjuvant CMF. *Ann Oncol* **8**: 271–275

Freeman SM, Abboud CM, Whartenby KA, Packmann CH, Koeplin DS, Moolten FL and Abraham GN (1993) The ‘bystander effect’: tumor regression when a fraction of the tumor mass is genetically modified. *Cancer Res* **53**: 5274–5283

Friedkin M and Roberts D (1953) The enzymatic synthesis of nucleosides. Thymidine phosphorylase in mammalian tissue. *J Biol Chem* **207**: 245–256

Grem JL and Fischer PH (1986) Alteration of fluorouracil metabolism in human colon cancer cells by dipyridamole with a selective increase in fluorodeoxyuridine monophosphate levels. *Cancer Res* **46**: 6191–6199

Griffiths L and Stratford IJ (1997) Platelet derived endothelial cell growth factor thymidine phosphorylase in tumour growth and response to therapy. *Br J Cancer* **76**: 689–693

Haraguchi M, Furukawa T, Sumizawa T and Akiyama S (1993) Sensitivity of human KB cells expressing platelet-derived endothelial cell growth factor to pyrimidine antimetabolites. *Cancer Res* **53**: 5680–5682

Huber BE, Austin EA, Good SS, Knick VC, Tibells S and Richard CA (1993) In vivo antitumor activity of 5-fluorocytosine on human colorectal carcinoma cells genetically modified to express cytosine deaminase. *Cancer Res* **53**: 4619–4626

Huber BE, Austin EA, Richards CA, Davis ST and Good SS (1994) Metabolism of 5-fluorocytosine in human colorectal tumor cells transduced with the cytosine deaminase gene: significant antitumor effects when only a small percentage of tumor cells express cytosine deaminase. *Proc Natl Acad Sci USA* **91**: 8302–8306

Ishitsuka H, Miwa M, Takemoto K, Fukuoka K, Itoga A and Maruyama HB (1980) Role of uridine phosphorylase for antitumor activity of 5′-deoxy-5-fluorouridine. *Jpn J Cancer Res* **71**: 112–123

Kato Y, Matsukawa S, Muraoka R and Tanigawa N (1997) Enhancement of drug sensitivity and a bystander effect in PC-9 cells transfected with a platelet-derived endothelial cell growth factor thymidine phosphorylase cDNA. *Br J Cancer* **75**: 506–511

Krenitsky TA (1968) Pentosyl transfer machanisms of the mammalian nucleoside phosphorylase. *J Biol Chem* **243**: 2871–2875

Lonn U, Lonn S, Nylen U and Winblad G (1989) 5-Fluoropyrimidine-induced DNA damage in human colon adenocarcinoma and its augmentation by the nucleoside transport inhibitor dipyridamole. *Cancer Res* **49**: 1085–1089

Luccioni C, Beaumatin J, Bardot V and Lefrancois D (1994) Pyrimidine nucleotide metabolism in human colon carcinomas: comparison of normal tissues, primary tumors and xenografts. *Int J Cancer* **58**: 517–522

Mader RM, Sieder AE, Braun J, Rizovski B, Kalipciyan M, Mueller MW, Jakesz R, Rainer H and Steger GG (1997) Transcription and activity of 5-fluorouracil converting enzymes in fluoropyrimidines resistance in colon cancer in vitro. *Biochem Pharmacol* **54**: 1233–1242

Miyadera K, Sumizawa T, Haraguchi M, Yoshida H, Konstanty W, Yamada Y and Akiyama S (1995) Role of thymidine phosphorylase activity in the angiogenic effect of platelet derived endothelial cell growth factor/thymidine phosphorylase. *Cancer Res* **55**: 1687–1690

Miyazono K, Okabe T, Urabe A, Takaku F and Heldin CH (1987) Purification and properties of an endothelial cell growth factor from human platelets. *J Biol Chem* **262**: 4098–4103

Moghaddam A and Bicknell R (1992) Expression of platelet-derived endothelial cell growth factor in *Escherichia coli* and confirmation of its thymidine phosphorylase activity. *Biochemistry* **31**: 12141–12146

Moolten FL (1986) Tumor chemosensitivity conferred by inserted herpes thymidine kinase genes: paradigm for a prospective cancer control strategy. *Cancer Res* **46**: 5276–5281

Moolten FL and Wells JM (1990) Curability of tumors bearing herpes thymidine kinase genes transferred by retroviral vectors. *J Natl Cancer Inst* **82**: 297–300

Mullen CA, Kilstrup M and Blaese RM (1992) Transfer of the bacterial gene for cytosine deaminase to mammalian cells confers lethal sensitivity to 5-fluorocytosine: a negative selection system. *Proc Natl Acad Sci USA* **89**: 33–37

Nishida M, Hino A, Mori K, Matsumoto T, Yoshikubo T and Ishitsuka H (1996) Preparation of anti-human thymidine phosphorylase monoclonal antibodies

 © 1999 Cancer Research Campaign useful for detecting the enzyme levels in tumor tissues. *Biol Pharm Bull* **19**: 1407–1411

O'Brien TS, Fox SB, Dickinson AJ, Turley H, Westwood M, Moghaddam A, Gatter KC, Bicknell R and Harris AL (1996) Expression of the angiogenic factor thymidine phosphorylase/platelet-derived endothelial cell growth factor in primary bladder cancers. *Cancer Res* **56**: 4799–4804

Parker WB, King SA, Allan PW, Lee Bennett Jr L, Secrist JA III, Montgomery JA, Gilbert KS, Waud WR, Wells AH, Gillespie GY and Sorscher EJ (1997) In vivo gene therapy with *E. coli* purine nucleoside phosphorylase. *Hum Gene Ther* **8**: 1637–1644

Patterson AV, Zhang H, Moghaddam A, Bicknell R, Talbot DC, Straford IJ and Harris AL (1995) Increased sensitivity to the prodrug 5′-deoxy-5-fluorouridine and modulation of 5-fluoro-2′-desoxyuridine sensitivity in MCF-7 cells transfected with thymidine phosphorylase. *Br J Cancer* **72**: 669–675

Rustum YM, Harstrick A, Cao S, Vanhoefer U, Yin MB, Wilke H and Seeber S (1997) Thymidylate synthase inhibitors in cancer therapy: direct and indirect inhibitors. *J Clin Oncol* **15**: 389–400

Schwartz EL, Baptiste N, O'Connor CJ, Wadler S and Otter BA (1994) Potentiation of the antitumor activity of 5-fluorouracil in colon carcinoma cells by the combination of interferon and deoxyribonucleosides results from complementary effects on thymidine phosphorylase. *Cancer Res* **54**: 1472–1478

Schwartz EL, Baptiste N, Wadler S and Makower D (1995) Thymidine phosphorylase mediates the sensitivity of human colon carcinoma cells to 5-fluorouracil. *J Biol Chem* **270**: 19073–19077

Schwartz EL, Wan E, Wang FS and Baptiste N (1998) Regulation of expression of thymidine phosphorylase/platelet derived endothelial cell growth factor in human colon carcinoma cells. *Cancer Res* **58**: 1551–1557

Shaw J, Smillie RH, Miller AE and MacPhee DG (1988) The role of blood platelets in nucleoside metabolism: regulation of thymidine phosphorylase. *Mutat Res* **200**: 117–131

Sobrero AF, Aschele C and Bertino JR (1997) Fluorouracil in colorectal cancer. A tale of two drugs: implications for biochemical modulation. *J Clin Oncol* **15**: 368–381

Sumizawa T, Furukawa T, Haraguchi M, Yoshimura A, Takeyasu A, Ishizawa M, Yamada Y and Akiyama S (1993) Thymidine phosphorylase activity associated with platelet-derived endothelial cell growth factor. *J Biochem* **114**: 9–14

Trinh QT, Austin EA, Murray DM, Knick VC and Huber BE (1995) Enzyme/prodrug gene therapy: comparison of cytosine deaminase/5-fluorocytosine versus thymidine kinase/ganciclovir enzyme/prodrug systems in a human colorectal carcinoma cell line. *Cancer Res* **55**: 4808–4812

Wei MX, Tamiya T, Chase M, Boviatsis EJ, Chang TKH, Kowall NW, Hochberg FH, Waxman DJ, Breakefield XO and Chiocca EA (1994) Experimental tumor therapy in mice using the cyclophosphamide-activating cytochrome P450 2B1 gene. *Hum Gene Ther* **5**: 969–978

Yoshimura A, Kuwazuru Y, Furukawa T, Yoshida H, Yamada K and Akiyama S (1990) Purification and tissue distribution of human thymidine phosphorylase; high expression in lymphocytes, reticulocytes and tumors. *Biochim Biophys Acta* **1034**: 107–113

Zimmerman M and Seidenberg J (1964) Deoxyribosyl transfer. Thymidine phosphorylase and nucleoside deoxyribosyltransferase in normal and malignant tissue. *J Biol Chem* **230**: 2618–2621

*© 1999 Cancer Research Campaign*
